# Red fluorescence in reef fish: A novel signalling mechanism?

**DOI:** 10.1186/1472-6785-8-16

**Published:** 2008-09-16

**Authors:** Nico K Michiels, Nils Anthes, Nathan S Hart, Jürgen Herler, Alfred J Meixner, Frank Schleifenbaum, Gregor Schulte, Ulrike E Siebeck, Dennis Sprenger, Matthias F Wucherer

**Affiliations:** 1 Faculty of Biology, University of Tübingen, Auf der Morgenstelle 28, 72076 Tübingen, Germany; 2 School of Biomedical Sciences, University of Queensland, Brisbane, Queensland 4072, Australia; 3 Department of Theoretical Biology, Faculty of Life Sciences, University of Vienna, Althanstrasse 14, 1090 Vienna, Austria; 4 Institute of Physical and Theoretical Chemistry, University of Tübingen, Auf der Morgenstelle 8, 72076 Tübingen, Germany

## Abstract

**Background:**

At depths below 10 m, reefs are dominated by blue-green light because seawater selectively absorbs the longer, 'red' wavelengths beyond 600 nm from the downwelling sunlight. Consequently, the visual pigments of many reef fish are matched to shorter wavelengths, which are transmitted better by water. Combining the typically poor long-wavelength sensitivity of fish eyes with the presumed lack of ambient red light, red light is currently considered irrelevant for reef fish. However, previous studies ignore the fact that several marine organisms, including deep sea fish, produce their own red luminescence and are capable of seeing it.

**Results:**

We here report that at least 32 reef fishes from 16 genera and 5 families show pronounced red fluorescence under natural, daytime conditions at depths where downwelling red light is virtually absent. Fluorescence was confirmed by extensive spectrometry in the laboratory. In most cases peak emission was around 600 nm and fluorescence was associated with guanine crystals, which thus far were known for their light reflecting properties only. Our data indicate that red fluorescence may function in a context of intraspecific communication. Fluorescence patterns were typically associated with the eyes or the head, varying substantially even between species of the same genus. Moreover red fluorescence was particularly strong in fins that are involved in intraspecific signalling. Finally, microspectrometry in one fluorescent goby, *Eviota pellucida*, showed a long-wave sensitivity that overlapped with its own red fluorescence, indicating that this species is capable of seeing its own fluorescence.

**Conclusion:**

We show that red fluorescence is widespread among marine fishes. Many features indicate that it is used as a private communication mechanism in small, benthic, pair- or group-living fishes. Many of these species show quite cryptic colouration in other parts of the visible spectrum. High inter-specific variation in red fluorescence and its association with structures used in intra-specific signalling further corroborate this view. Our findings challenge the notion that red light is of no importance to marine fish, calling for a reassessment of its role in fish visual ecology in subsurface marine environments.

## Background

**Figure 1:**
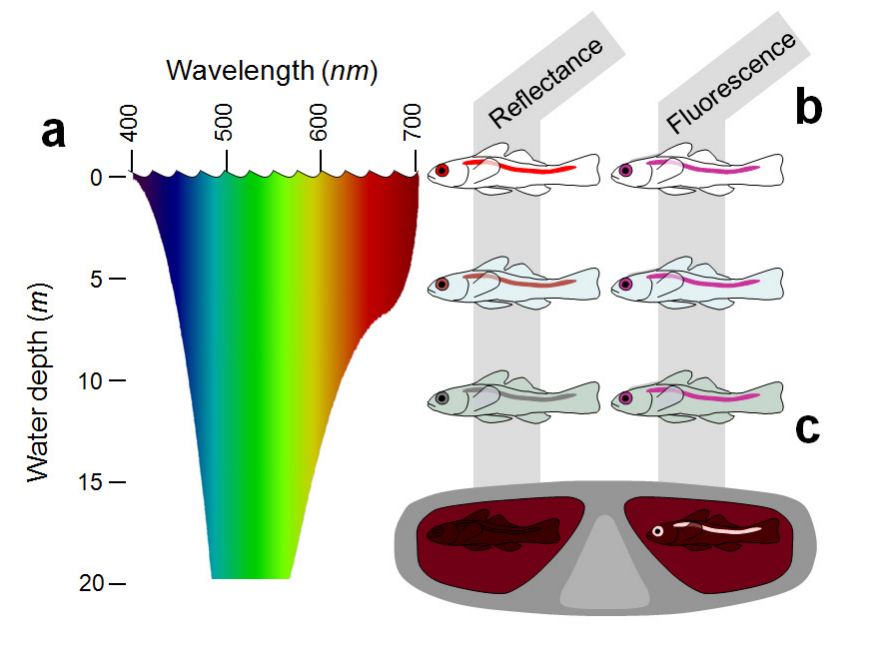
**General introduction to light attenuation and observation of natural fluorescence in near-shore marine environments**. **a**. The visual spectrum ranges from 400 to 700 nm at the water surface, but downwelling sunlight loses the red component (600–700 nm) rapidly within 10–15 m (modified from Pinet PR (2000) Invitation to Oceanography. Jones and Bartlett). UV and violet wavelengths are attenuated less rapidly. The attenuation with depth of spectral composition (and light intensity, not shown) varies strongly with the concentration of organic matter in the water column. **b**. Most red pigmentation is based on reflectance of the red component of ambient light and therefore only appears "red" when close to the surface during daytime or under broad spectral light (e.g. dive torch). Fish with this pigmentation appear dull grey in deeper water. Red fluorescent patterns, however, continue to appear reddish and bright, even in deeper water, where excitation of fluorescent pigments by shorter wavelengths induces redness. Note that red fluorescence is rarely perceived as pure red, but is mostly an enhancer of mixed colours such as pink, lilac or red brown. Even so, it remains clearly visible in deeper water as a contrast enhancer. Closer to the surface, fluorescent patterns are masked by reflective colouration (e.g. yellow and red in *Eviota pellucida*, Fig. 3). **c**. Since excitation frequencies (blue-green) are brighter than emission frequencies (red in our example) red fluorescence is best seen when viewed through a filter that blocks the excitation frequencies and only allows the emission frequencies to pass. When looking through a red filter in e.g. 20 m depth, all remaining red light must be "locally produced" through fluorescence or bioluminescence. Given that fluorescence exploits light energy from ambient light, it is more efficient than bioluminescence and therefore likely to be the mechanism of choice for diurnal fish.

At depths below 10 m, reefs are dominated by blue-green light because seawater selectively absorbs the longer, 'red' wavelengths (600 nm and more) from downwelling sunlight (Fig. 1)[1,2]. Consequently, many reef fish have visual pigments matched to shorter wavelengths, which are transmitted better by water [3-5]. In addition, ecological studies of fish vision must correct for the spectrum available at the depth where they live [1,6,7] and therefore routinely correct spectral sensitivity measurements from the laboratory for the available (mostly downwelling) light on the reef. This reduces the relevance of red light to reef fish even more. However, this procedure ignores the fact that several marine organisms, including deep sea fish, produce their own red bioluminescence and are capable of seeing it [8,9]. The purpose of this study was (1) to see "with our own eyes" whether there is indeed a lack of red light at depth in the euphotic zone during daytime and (2) to identify the observed sources of natural red fluorescence in fish in particular. This work combines results from several studies carried out on coral reefs in the Red Sea and the Great Barrier Reef and has been supplemented by observations and measurements on fish in the laboratory.

## Results

### Seeing red fluorescence on reefs

**Figure 2:**
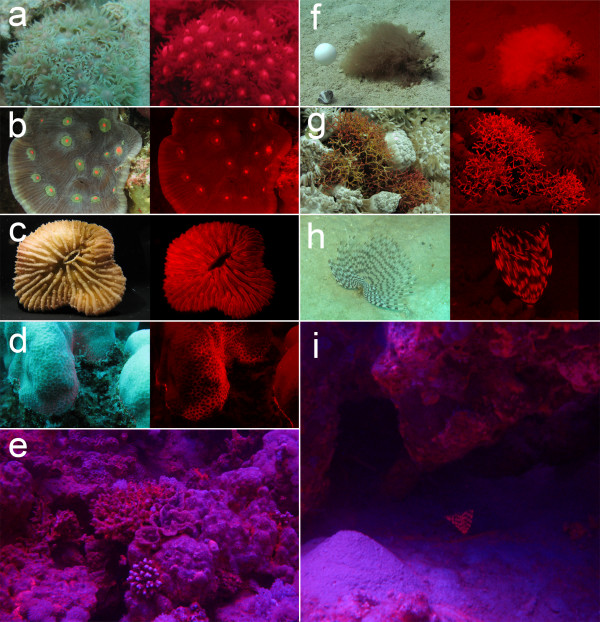
**Examples of common red fluorescent invertebrates on coral reefs**. **a-d**. Stony corals (**a**. *Goniopora*, **b**. *Mycedium*, **c**. *Fungia*, **d**. *Porites*). **e**. Reef scenery, as seen through a Lee Medium Red filter. **f**. Unidentified alga (white pingpong ball as reflectance reference). **g**. Calcareous alga *Amphiroa*. **h**. Polychaete worm *Sabellastarte indica*. **i**. Typical environment of *S. indica *under reef ledge. Pictures **a-d **and **f-h **show object under natural illumination (left) and as seen through a red filter (right). Pictures **e **and **i **show fluorescence in the field as seen by a digital camera. Most pictures taken in the field (Dahab, Egypt) under natural illumination between 14 and 17 m depth. Only **c **was photographed in the laboratory. Other reef invertebrates seen to fluoresce were sponges (e.g. *Aaptos*, *Acanthella*, *Theonella*) and feather stars (e.g. *Colobometra*, *Oligometra*).

We separated excitation from emission wavelengths in the field under natural, day-time solar illumination by SCUBA-diving below the penetration depth of the red component of sunlight (15–30 m) using masks and cameras equipped with a red filter blocking wavelengths below 600 nm (Fig. 1). This revealed widespread, natural red fluorescence produced by many microorganisms, plants and invertebrates (Fig. 2, see Additional file 1). The latter included mostly corals [10-13], but also species for which red fluorescence has never been described before, such as a polychaete species, several sponges (not shown) and feather stars (details in Fig. 2).

### Red fluorescent fishes

**Figure 3:**
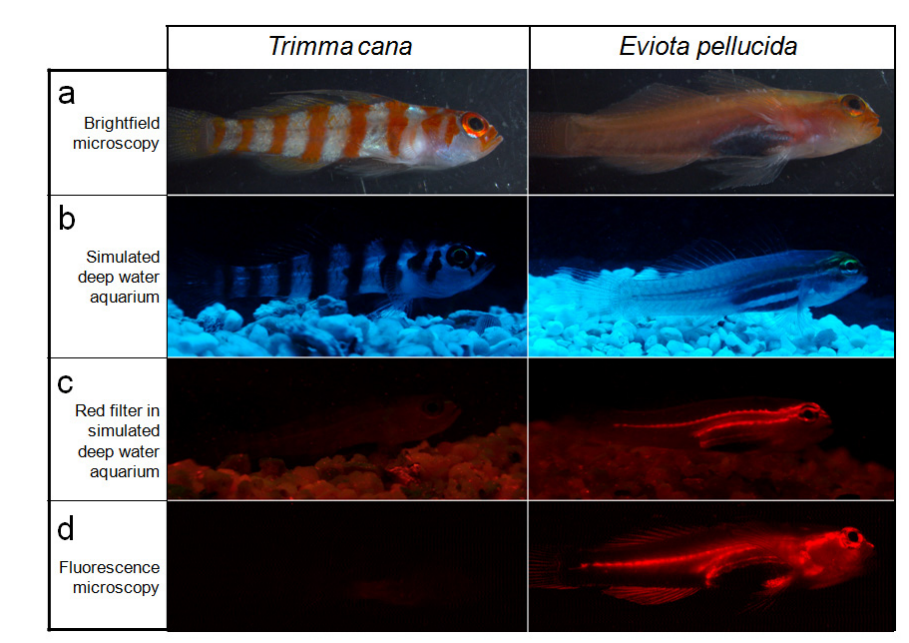
**How to distinguish a red fish from a red fluorescent fish?**. Comparison between a non-fluorescent goby, *Trimma cana *(left), and a similar sized, red fluorescent goby, *Eviota pellucida *(right) under four viewing conditions. **a**. Artificial white light from a strong Schott KL 2500 LCD halogen cold light source under a Leica stereomicroscope (MZ 16F). **b**. In a halogen-illuminated aquarium with downwelling light filtered through Lee 729 Scuba-Blue filter (transmission range 400–550 nm, λ_max _= 500 nm), thus simulating light at depth. **c**. Illumination as in **b**, but viewed through a red filter, revealing red fluorescence. **d**. Illumination as in **a**, seen under a Leica fluorescence stereomicroscope (MZ 16F) using green light for excitation, while viewing through red filter. The differences between the viewing conditions illustrate that red fluorescence can only be reliably seen when excitation and emission frequencies are separated, as at depth in the sea or under blue light, and by using a red filter for viewing.

**Figure 4:**
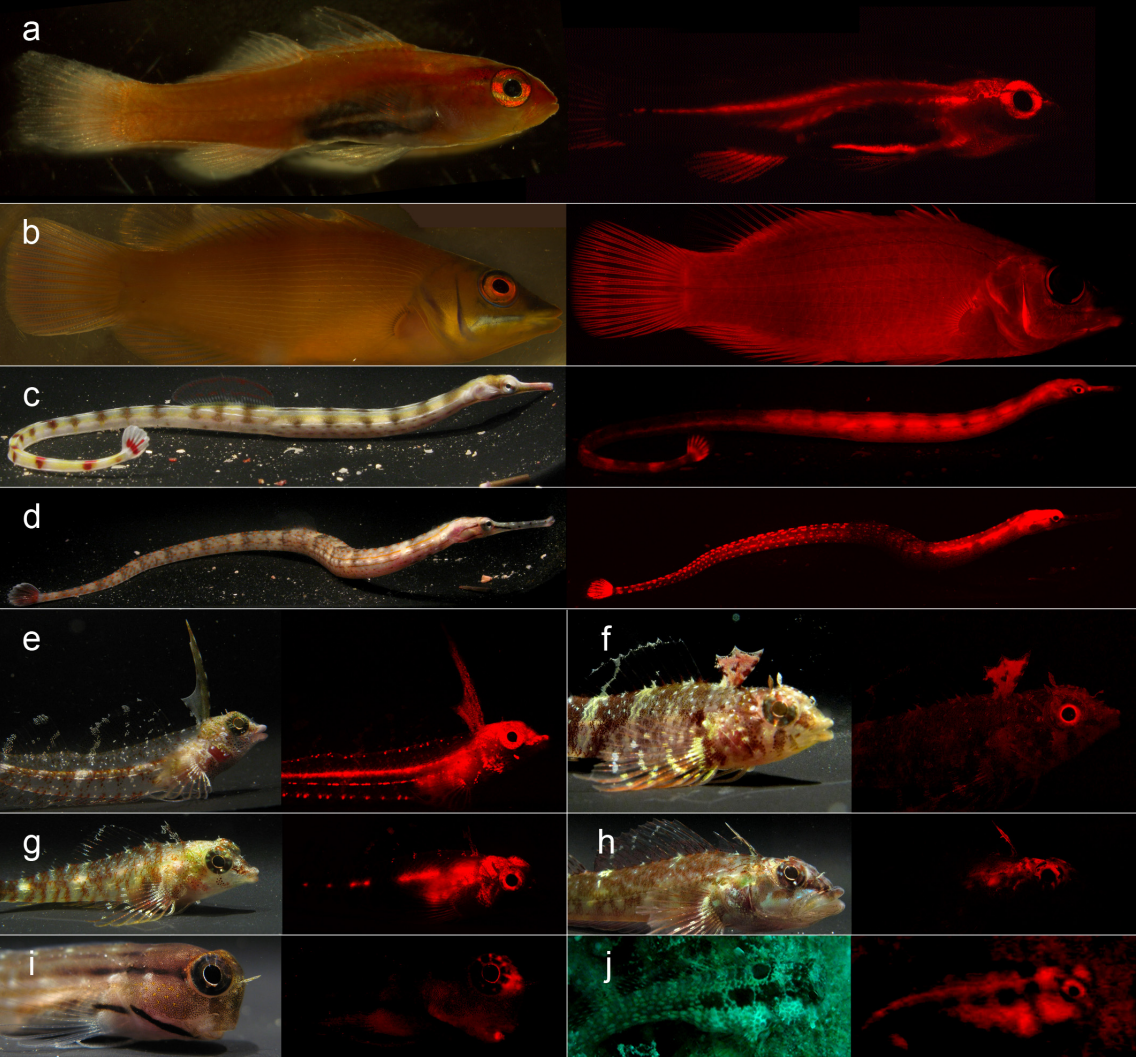
**Red fluorescent representatives of five different reef fish families**. **a**. *Eviota pellucida *(Gobiidae). **b**. *Pseudocheilinus evanidus *(Labridae). **c**. *Corythoichthys flavofasciatus *and **d**. *C. schultzi *(Syngnathidae), **e**. *Enneapterygius pusillus*, **f**. *E. destai*, **g**. *E. abeli *and **h**. *Helcogramma steinitzi *(Tripterygiidae). **i**. *Ecsenius dentex *and **j**. *Crossosalarias macrospilus *(Blenniidae). All pictures are from the laboratory, except for **j **(field). Left: broad spectrum illumination, right: red fluorescence under blue (laboratory) or natural (field) illumination.

**Figure 5:**
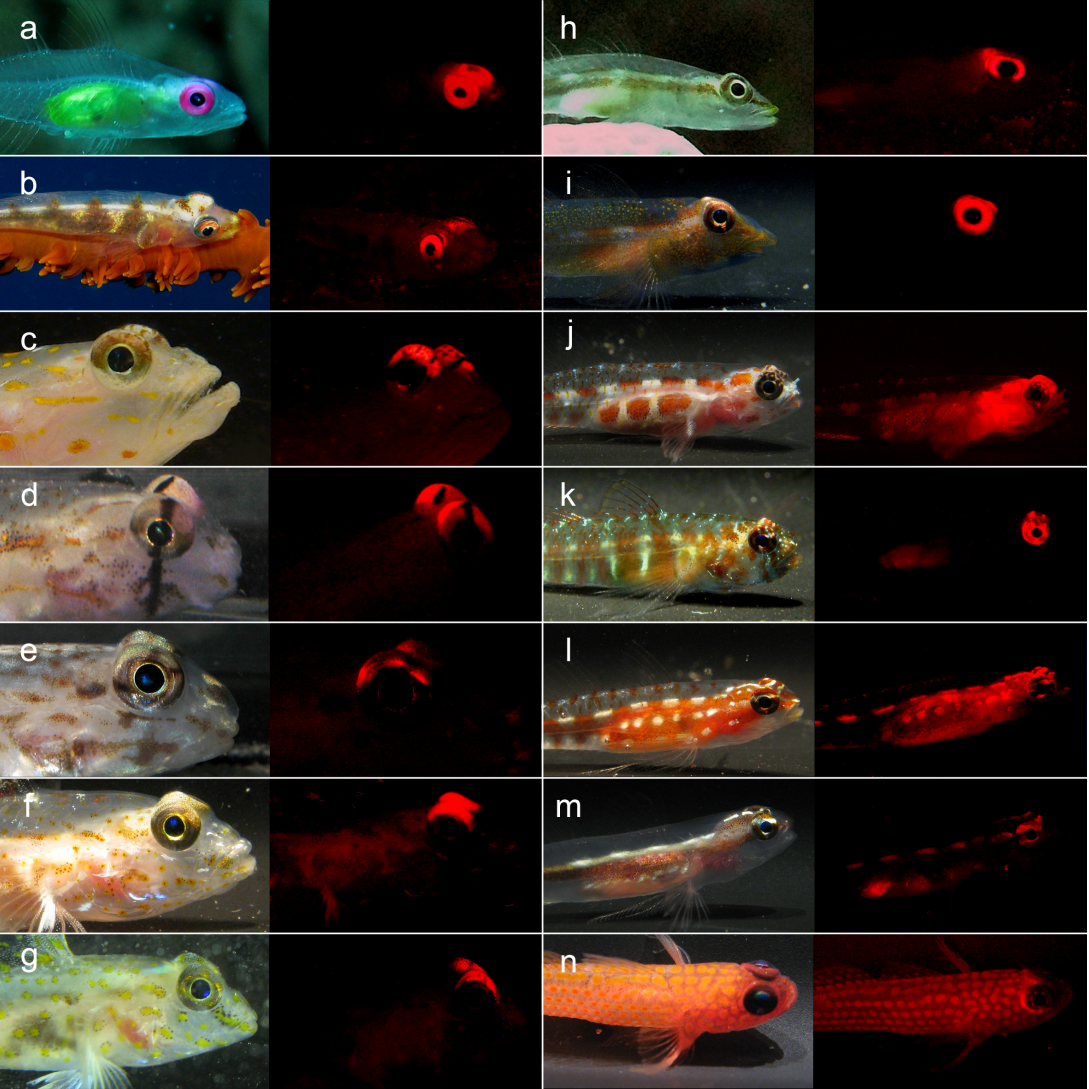
**Diversity in red fluorescence in 14 goby species**. **a**. *Bryaninops natans*. **b**. *B. yongei*. **c**. *Ctenogobiops tangaroai*. **d**. *Gnatholepis anjerensis*. **e**. *Istigobius decoratus*. **f**. *Fusigobius duospilus*. **g**. *F. longispinus*. **h**. *Pleurosicya micheli*. **i**. *P. prognatha*. **j**. *Eviota guttata*. **k**. *E. prasina*. **l**. *E. zebrina*. **m**. *E. sebreei*. **n**. *Trimma avidori*. All fish shown under broad spectrum illumination (left) in the laboratory (halogen) or field (a, b and h) and under blue illumination (right) with red filter.

**Figure 6:**
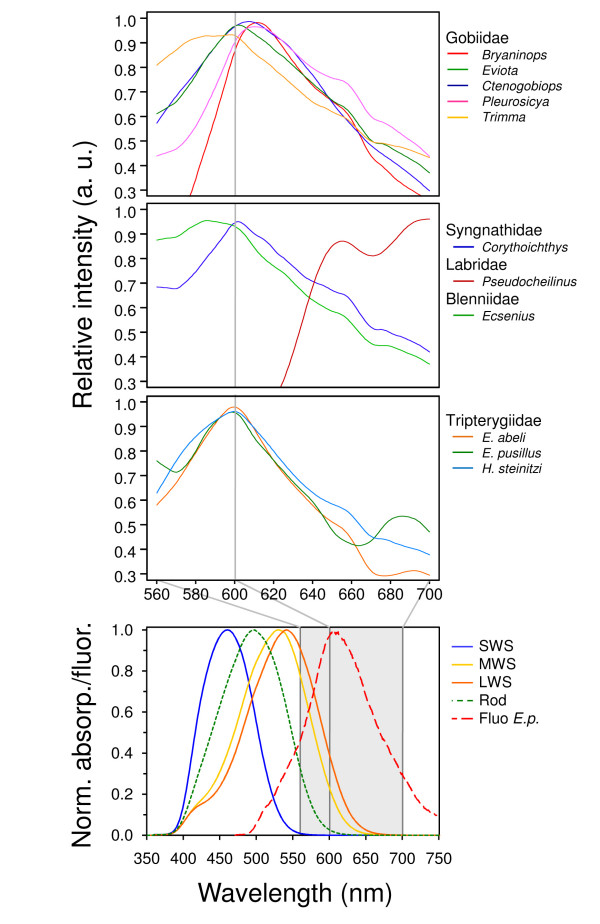
**Spectrometric measurements confirm the emission of red light, and the ability to see it**. **a**. Fluorescence emission spectra of five genera of Gobiidae (top), one genus of Syngnathidae, Labridae and Blenniidae each (middle) and three species of Tripterygiidae (bottom). **b**. Absorptance spectra of photoreceptor visual pigments found in *Eviota pellucida *with wavelengths of maximum absorptance (λmax) at 497 (rods), 458 (SWS single cones), 528 (MWS, twin cones) and 540 nm (LWS, twin cones). The fluorescence emission spectrum of *E. pellucida *is included for comparison (dashed line).

Of central importance here is our discovery of red fluorescence in reef fishes. Using the principle described above to distinguish "regular" red colouration from red fluorescence (Fig. 3) we identified at least 32 fish species belonging to 16 genera in 5 families that fluoresced visibly in red (Fig. 4, 5, Table 1, see also Additional file 2). Fluorescent patterns usually included the eye ring and parts of the head or thorax and varied substantially between congeners (e.g. in the genera *Eviota *or *Enneapterygius*). Fins rarely fluoresced, except for the anal fin (some Gobiidae), the first dorsal fin (Tripterygiidae) or the tailfin (Syngnathidae). A 'whole body glow', including all fins, was present in the small wrasses *Pseudocheilinus evanidus *and *Paracheilinus octotaenia*. Visual and photographic evidence from the field was confirmed by extensive fluorescence microscopy and spectrometry of representative cases (Table 1). Fluorescence showed peak emission around 600 nm in most species (Fig. 6a, Table 1). *Enneapterygius pusillus *showed a second small peak at around 680 nm. *P. evanidus *differed from all others by having a double peak at 650 and 700 nm. We tested various light sources, including UV, but were not able to detect fluorescent emission at other (shorter) wavelengths in the fish described here. Anecdotal field observations suggest that yellow fluorescence may be present in other fishes (pers. obs.).

**Table 1:** Reef fish species that were found to show red fluorescence

Family	Species	Site	Size (cm)	Red fluorescent body parts	Strength	Guanine crystals	Peak emission (nm)
**Syngnathidae**							
	*Corythoichthys flavofasciatus*	2	12	1, 2, 4, 8, 7	+++	yes	599–604
	*C. schultzi*	2	15	1, 2, 4, 7, 9	+++	yes	600–605
**Labridae**							
	*Paracheilinus octotaenia*	1, 2	9	4	++	-	653–658, 695–699
	*Pseudocheilinus evanidus*	1, 2, 4	8	4	++	no	-
**Blenniidae**							
	*Crossosalarias macrospilus*	3	8	1, 2, 4, 10	+++	-	-
	*Ecsenius dentex*	2	6	1, 2	+	yes	584–589
**Tripterygiidae**							
	*Enneapterygius abeli*	2	2.5	1, 2, 4	++	-	597–602
	*E. destai*	2	2	1, 5	++	-	-
	*E. mirabilis*	3	3.5	1, 5, 8, 10	+++	-	-
	*E. pusillus*	2	2	1, 2, 4, 5, 8, 10	+++	yes	596–601, 684–689
	*Helcogramma steinitzi*	2	5	1, 2, 5	+++	-	597–602
	*Ucla xenogrammus*	3	5.5	1, 10	+++	-	-
**Gobiidae**							
	*Bryaninops natans*	1, 2, 5	2.5	1	+++	yes	605–610
	*B. ridens*	2, 5	2	1	+	-	-
	*B. yongei*	2, 5	2.5	1	++	-	-
	*Ctenogobiops maculosus*	1, 2	7	1	+++	-	-
	*C. tangaroai*	4	7	1	+++	yes	605–610
	*Eviota distigma*	2	2	1, 11	+	-	too weak
	*E. guttata*	2	2.5	1, 2, 4, 6, 9	+++	-	605–610
	*E. nigriventris*	4	2.5	1, 2	+	-	-
	*E. pellucida*	3, 4	2.5	1, 2, 3, 6	+++	yes	604–609
	*E. prasina*	2	3	1, 12	+	-	596–601
	*E. queenslandica*	3	3	1	+		-
	*E. sebreei*	2	2.5	1	+	yes	585–590
	*E. zebrina*	3, 5	3	1, 2, 4, 9	+++	yes	600–604
	*Fusigobius duospilus*	2	6	1	+++	-	-
	*F. longispinus*	2	7	1	+	yes	600–605
	*Gnatholepis anjerensis*	2	8	1	+	yes	-
	*Istigobius decoratus*	2	12	1	+	yes	591–596
	*Pleurosicya micheli*	2, 5	3	1	++	yes	601–606
	*P. prognatha*	2, 5	2	1	+++	yes	608–613
	*Trimma avidori*	2	3	1, 2, 9	+	-	590–595

Study localities: 1 = El Quseir (Sharm Fugani, Egypt, field); 2 = Dahab Marine Research Centre (South Sinai, Egypt, field and laboratory), 3 = Lizard Island Research Station (Queensland, Australia, field and laboratory), 4 = Tübingen (laboratory), 5 = Collection JH, Vienna. Fluorescent body parts are encoded as follows: 1 = Eye rings, 2 = Head (dorsal), 3 = Lateral lines, 4 = Trunk, 5 = First dorsal fin, 6 = Anal fin, 7 = Tailfin, 8 = Snout, 9 = Dots on body, 10 = Spine, 11 = Gut. Strength of fluorescence is a consensus measure among observers based on visibility in the field and/or the laboratory: Strong (+++) means strikingly bright in the field as well as in the laboratory, easy to photograph and strong spectrometric signal; weak (+) means only visible under laboratory conditions, requiring long integration times for fluorescence measurement and long exposure times for photography (optimized for each specimen). Peak emission is shown as 5 nm interval.

### Mechanism of red fluorescence in fishes

**Figure 7:**
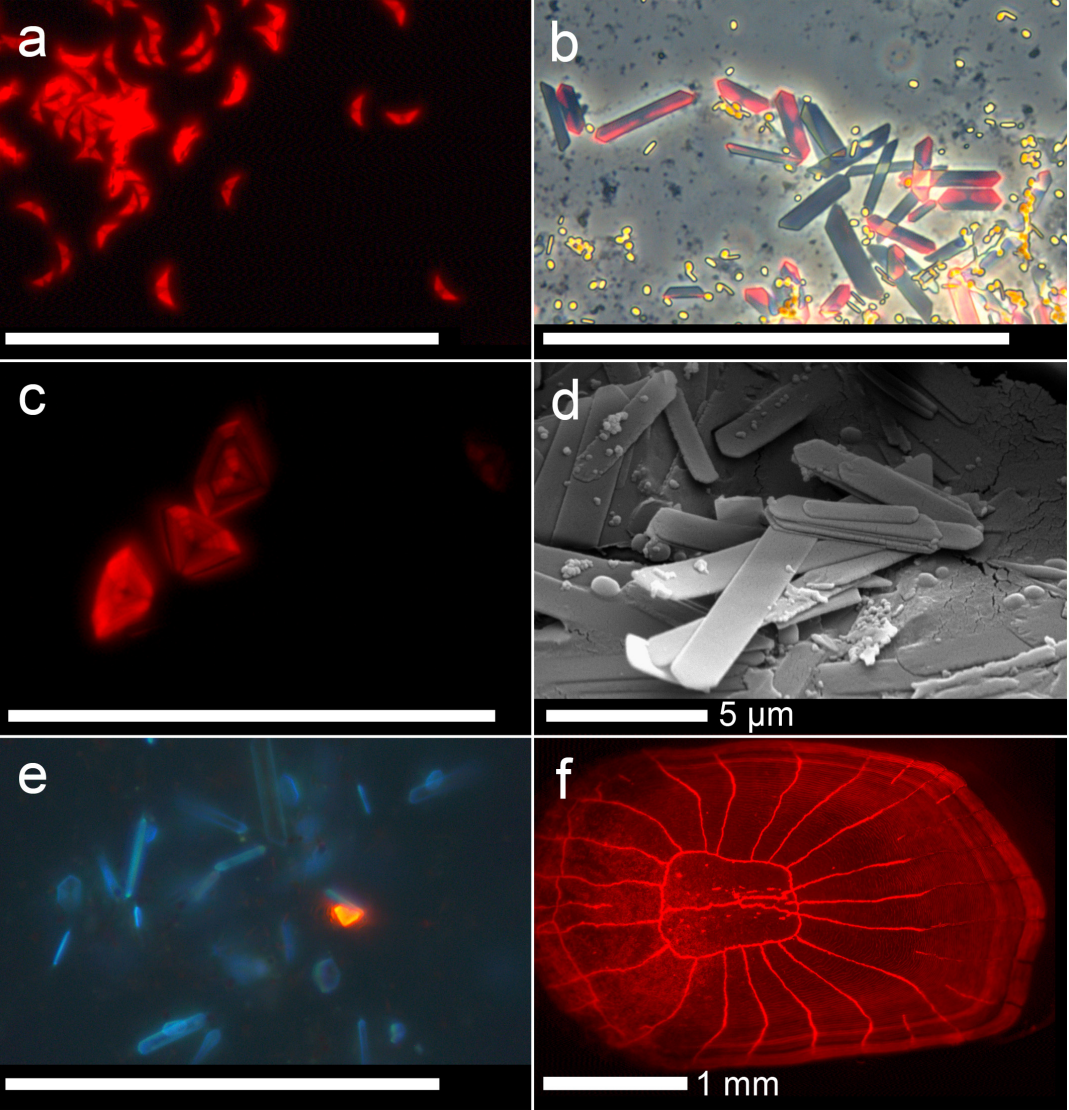
**Sources of red fluorescence in fishes**. **a**. Guanine crystals from the eye ring of *Eviota pellucida *(fluorescence microscopy). **b**. Same from *Ctenogobiops tangaroai *(fluorescence overlaid with phase contrast). **c**. As in **b**, diamond form (fluorescence microscopy). **d**. Guanine crystals falling apart in characteristic platelets (from eye ring of *E. pellucida*, scanning electron micrograph). **e**. Single red fluorescent guanine crystal among normal crystals in eye ring of the non-fluorescent goby *Trimma cana *(fluorescence microscopy, see also Fig. 3). **f**. Scale of *Pseudocheilinus evanidus*, in which the red fluorescent pigment is associated with bony scales and fin rays (fluorescence microscopy). Scale bar = 50 μm unless indicated otherwise.

Dissection revealed that red fluorescence was associated with guanine crystals in pipefish, triplefins, blennies and gobies (Fig. 7, Table 1). Guanine crystals are produced by iridophores and are well known as the source of silvery reflection and iridescence in bony fish[14]. However, they have never been described to show strong red fluorescence. In *E. pellucida *and *Ctenogobiops tangaroai*, only about half of the crystals isolated from the eye rings showed bright red fluorescence (Fig. 7b), suggesting that the fluorescing substance is produced or sequestered independently from the crystals. Preparations of crystals maintained strong fluorescence after prolonged storage in a dried or liquid form (70% EtOH or 4% formaldehyde), allowing us to confirm fluorescence in preserved gobies collected up to 5 years before (Collection JH, Vienna, Table 1). This is in striking contrast to reflective red pigmentation, which bleaches out within hours after fixation. We did not find fluorescent guanine crystals in *P. evanidus*. Here, microscopic investigation suggests the presence of a fluorescent pigment associated with the bony tissue of scales and fin rays (Fig. 7f). This pigment has a chemical stability in preserved specimens that matches that of guanine-linked red fluorescence.

As a control, we isolated guanine crystals from non-fluorescing fish. In *T. cana *(Fig. 3), *Psetta maxima *(turbot) and *Engraulis encrasicolus *(anchovy), we found less than 1% of the crystals to fluoresce in red (Fig. 7e), suggesting that a very low level of red fluorescence may be widespread and is not limited to reef fish.

### Can red fluorescent fish see red fluorescence?

With few exceptions [15-17] most marine fishes lack a sensitivity reaching into the red part of the spectrum[5]. But as far as we know, none of the fish genera listed in Table 1 has ever been tested for its spectral sensitivity. Here, we measured the retinal spectral sensitivity of wild-caught *E. pellucida*. Their retina contains rods and both single and twin cones as previously found in one other goby[18]. Mean wavelengths of maximum absorbance (λ_max_) of the visual pigments in the rods and short-wavelength-sensitive (SWS) single cones were 497 and 458 nm, respectively. λ_max _values in the twin cones were highly variable, ranging from 518–546 nm, although spectra were clustered into two groups with mean λmax values at 528 (medium-wavelength-sensitive, MWS) and 540 nm (long-wavelength-sensitive, LWS; Fig. 6b). The majority of twin cones had the 528 nm pigment in both members (528/528 twins) although both 528/540 and 540/540 twins were also observed. These results suggest that there may be more than two distinct visual pigments in the twin cones, and/or co-expression of opsin genes within the same outer segment, as in other fish[19]. Regardless, Fig. 6b shows that there is considerable overlap between the red fluorescence emission spectrum and the absorbance spectra of the visual pigments in the twin cones. It is highly likely, therefore, that this species can see its own fluorescence. A similar result is expected for *Corythoichthys *pipefish, given that long wavelength sensitivity is known from other Syngnathids [17].

## Discussion

### The function of red fluorescence

Why do some reef fish fluoresce strongly in red, whereas most do not? Although non-fluorescent (reflective) red pigmentation is widespread in reef fish[20], it appears grey or black at depth (Fig. 3), allowing fish to blend in with their background[21]. Red fluorescence, however, does the opposite: by emitting a colour that is lacking from the environment, a fish contrasts more against its background. As a result, red fluorescence may function as a communication or attraction signal, as proposed for red-bioluminescent deep sea fishes[16] and siphonophores[22], ultraviolet-reflecting reef fishes[23] and green fluorescent mantis shrimp[24] and parrots[25].

**Figure 8:**
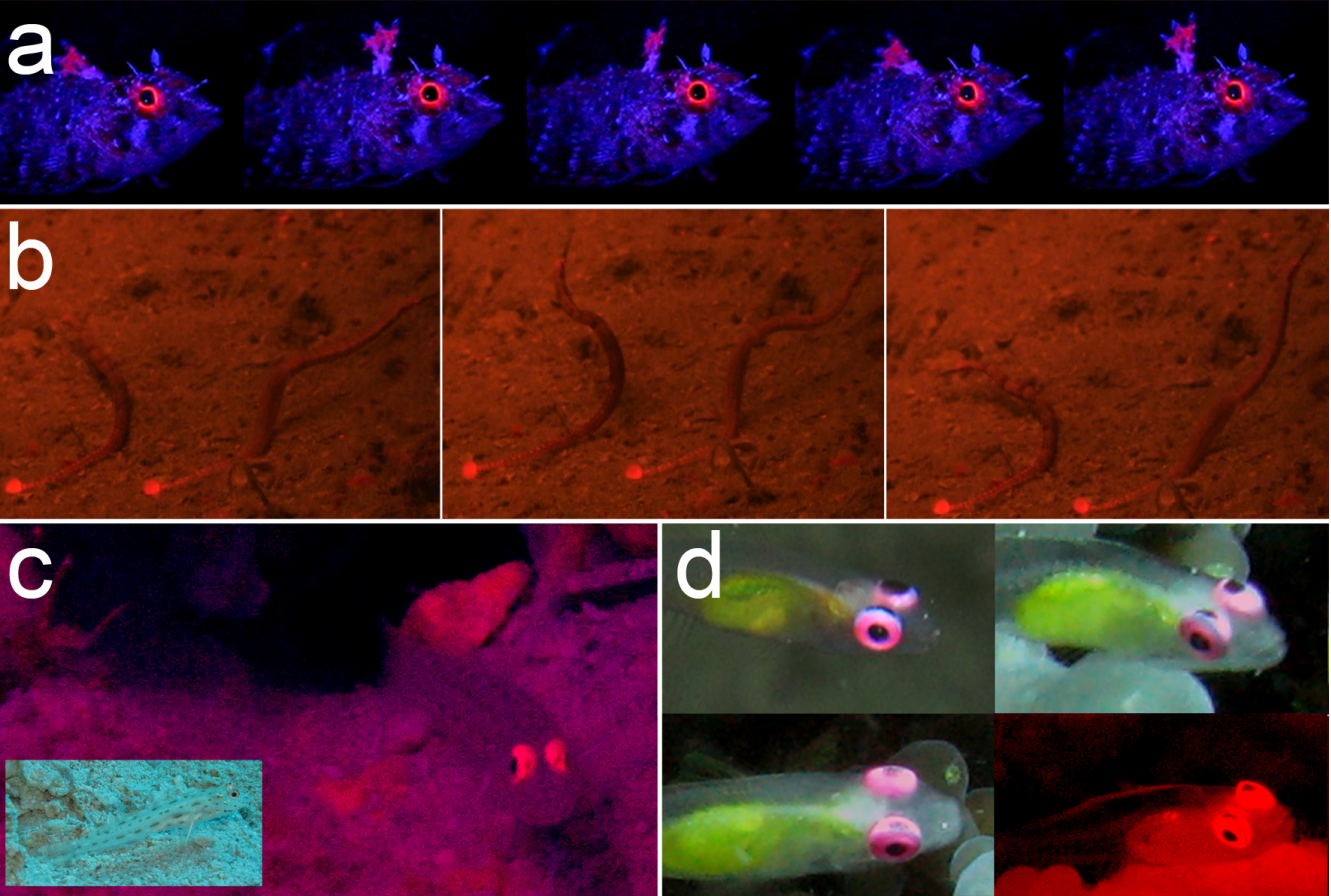
**Red fluorescence in a signalling context**. **a-b**. Frameshots of two videos suggestive of communication involving fluorescent fins. **a**. *Enneapterygius destai *waving its first dorsal fin when 'excited' (laboratory, from Additional file 3, sequence 2b). **b**. *Corythoichthys schultzi *pair interacting, displaying conspicuous red fluorescence on the tail plate (natural reef illumination, 20 m depth, from Additional file 3, sequence 1). **c**. *Ctenogobiops maculosus *is cryptic when sitting at its burrow entrance under natural illumination (insert), but shows conspicuous eyes when emphasizing red wavelengths (Additional file 2). **d**. *Bryaninops natans *with a pupil-like black spot on the upper part of the fluorescent eye ring suggesting that fluorescence and (deceptive) gaze signalling may be linked in this species (Additional file 2).

We see four reasons why red fluorescence may be part of a private communication system in fish. Firstly, peaking mostly around 600 nm, red fluorescence is at the borderline of what is visible to many marine fishes, and due to rapid attenuation of red light by water, even those that can see red will be able to see it over short distances only. This is suggestive of an adaptive shift away from the "public area" of the visual spectrum into a bordering "private area". Secondly, most species found to fluoresce are small, benthic, pair- or group-living fishes, often with conspicuous intra-specific behaviours, but quite cryptic colouration in other parts of the visible spectrum. Thirdly, there is strong inter-specific variation within and between closely related genera suggestive of species-recognition (Fig. 4, 5). Finally, fluorescence is present in structures that are used in intra-specific signalling. This is true for the first dorsal fin in triplefins (Fig. 8a, Additional file 3, sequence 2b), the tailfin in pipefish (Fig. 8b, Additional file 3, sequence 1) and possibly the anal fin in *E. pellucida *(Fig. 3) and *E. guttata*. Fluorescent eye rings may function as an indicator of presence (Fig. 8c) or reveal the direction of gaze (Fig. 4e–g, Fig. 8a, d, Additional file 3, sequence 2b). Since iridophores are involved in rapid colour change in other fishes [26,27], fluorescent fish may also be able to change the strength of fluorescence.

One reason why red fluorescence in fishes may have escaped attention is because fluorescence is usually observed during night dives using strong UV light sources. Although this reveals the spectacular bluish-green fluorescence typical of many corals, it is not a good strategy to visualize red fluorescence because excitation and emission wavelengths are far apart, making red fluorescence weak relative to the shorter wavelength emissions. Moreover, most fish are hiding at night, explaining why red fluorescence had not yet been described for fishes. Up till now we have not yet found a nocturnal fish that fluoresces in red, which fits well with widespread reflectant red colouration indicating that crypsis is more important in these species.

### Final remarks

For a correct interpretation of our results, it is important to stress that it is not required to look through a red filter to "see" red fluorescence during daytime, particularly not for the stronger cases. Using a filter merely facilitates its detection in the field. Without a filter, red fluorescence at depth is usually only one element in complex, multi-spectral, red-containing colours, such as orange-brown, red-brown, pink, lilac, violet or even bright white in some encrusting corals. Cases of pure fluorescence-based red are thus far limited to a few corals and sponges. Whether pure or mixed, red-containing colours cannot be generated by reflectance and therefore sources of red fluorescence can be identified without a filter at depth. This merely requires special attention by the diver: Although e.g. red-brown is prevalent on reefs, it is not perceived as unusual or unexpected by most. Consequently, while red masks are invaluable for initial detection, fluorescence photography is easiest using a regular mask and a camera with a red filter. An alternative is to refrain from a red filter altogether and to adjust the white balance of the camera manually to local light conditions using a white slate. This will emphasize reds, while suppressing, but not removing, shorter wavelengths. At depth, this will highlight any red fluorescence. Applying this to fish colour vision, we suspect that, if red fluorescent fish can indeed see their fluorescence, they see it as enhanced contrast involving pink, red-brown or other red-containing, mixed colours in an otherwise blue-green environment. Consequently, behavioural experiments should not test the ability of fish to see weak red light, but their ability to distinguish between multi-spectral signals with and without a weak red component in a blue-green flooded environment.

Finally, we want to make a cautionary remark on diving safety. Diving with a red mask is similar to night diving, with dramatically reduced light intensities and viewing distances. Disorientation becomes a serious problem. Moreover, it takes several minutes to adapt to the darkness. Staying in a small, familiar area and moving slowly and carefully is crucial. Furthermore, it is essential to take a torch to read equipment. Dials, indicators and computer backlights are either reflectant or luminesce blue or green, making them effectively illegible at depth in the absence of a local white (red-containing) light source. To circumvent this problem, we also used the Oceanic DataMask which has a built-in dive computer that can be read irrespective of any filter attached to the front. Because of these unfamiliar restrictions, we recommend that only experienced divers use this procedure and that only one partner in a buddy team uses a red mask at any given time. We also recommend attaching filters in such a way that they can be instantly removed without having to change to a spare mask, which one should carry nevertheless.

## Conclusion

We conclude that a considerable number of reef fishes have developed complex patterns of striking red fluorescence which may be used to enhance visual communication by exploiting a waveband invisible to most other fish. At least one species (*E. pellucida*) shows a retinal sensitivity to its own red fluorescence and many species show suggestive evidence that fluorescence is linked to signalling structures. Additional studies are required to confirm that this ability is also present in other fluorescent fish. Nevertheless, the prevalent assumption that red light is of low importance for reef fish[5] must be questioned. Obviously, a lack of downwelling red light is not a reason to stop seeing red, adding an exciting novel dimension to reef light ecology.

## Methods

### Material

Fish were collected in Dahab (Dahab Marine Research Centre, Egypt, with permit from NCS/EEAA) and at Lizard Island Research Station (Australia) (GBRMPA permit G05/13668.1 to UES) by anaesthetising individuals with clove oil (10 ml in 50 ml EtOH added to 200 ml sea water). Fish for laboratory work in Tübingen were obtained from the sustainable aquarium trade and kept in accordance with German animal care legislation. Species were identified using standard[20] and specialised [28-30] literature.

### Digital photography

Underwater pictures of fluorescence under natural illumination were taken with a Canon PowerShot G7 or G9, Canon Ixus 750 and Sony HD HDR-CX6EK using Lee Medium Red filters or Edmund Optics Y59-642 glass filters. Macro photography in the laboratory was done using Canon PowerShot G7 or G9 with blue LED light source (LUXEON K2 LXK2-PB14-P00, λmax = 470 nm) and Edmund Optics Optical filter G43-943. All of these filters block most light below 600 nm, but allow some blue or green light to leak through (e.g. Fig. 4a–b). This blue-green hue was removed in most pictures by only showing the red channel (590–700 nm) of the RGB digital image. Other pictures were taken using a Leica MZ16F fluorescent stereo-microscope on anaesthetised, freshly killed or preserved animals. A Leica DM5000 fluorescence microscope was used to document guanine crystals.

### Fluorescence spectrometry

Fluorescent emission spectra were recorded using an AvaSpec-2048-USB2 spectrometer with a green laser (λ_exc _= 532 nm) as excitation light source, yielding reproducible emission spectra of from live and anaesthetised fish. We supplemented these measurements with high-sensitivity spectrometry of fluorescent guanine crystals and scales of *C. tangaroai*, *E. pellucida*, *E. sebreei*, *E. zebrina*, *F. longispinus*, *I. decoratus*, *P. micheli *and *P. evanidus*. Here, emission spectra were induced using λ_exc _= 473 nm laserlight (LDH – P400B Picoquant) on a custom-built Zeiss Axiovert 135 TV confocal laser scanning microscope equipped with an avalanche photodiode (APD, SPCM-AQR-14, Perkin Elmer) and a spectrometer with cooled CCD camera (Spec-10:100B, Princeton Instruments). Since these measurements coincided well with those obtained with the AvaSpec-2048-USB2, data from both methods were pooled (Fig. 6). Spectra were normalized by setting maximum emission equal to 1. Curves for genera (Fig. 6) are averages of the average spectra of each available species.

### Scanning electron microscopy

For electron scanning micrographs, guanine crystals were isolated[14] from *E. pellucida*, coated with 20-nm Au/Pd and photographed using a Cambridge Stereoscan 250 Mk2 electron microscope (Fig. 7d).

### Retina microspectrometry

For microspectrometry of *E. pellucida *visual pigments, five gobies (13–18 mm standard length) were dark adapted for at least 1 hour and their retinas removed under infrared illumination with the aid of image converters. Each retina was dispersed mechanically and mounted in 340 mOsm kg-1 phosphate-buffered saline containing 10% dextran. Absorbance spectra (325–800 nm) of visual pigments housed in individual photoreceptor outer segments were measured using a wavelength-scanning microspectrophotometer, as described elsewhere[31] (Fig. 6b).

## Authors' contributions

NKM is the principal investigator and initiator and has been involved all aspects except for data collected in Australia. NA, JH, GS, DS and MFW collected field and laboratory data and contributed to data analysis and manuscript writing. GS was also responsible for spectrometry and digital recording. NSH and UES made the microspectrometric measurements of *E. pellucida *eyes and edited the manuscript. FS and AJM carried out the high-resolution spectrometry and provided technical background on fluorescence spectrometry. All authors have read and approved the final manuscript.

## Supplementary Material

Additional File 1 **Red fluorescent invertebrates**. Short MPG video clip showing examples of strongly fluorescent invertebrates and plants. **Sequence 1a: **Calcareous alga (*Amphiroa*) under normal viewing conditions (field at ca. 18 m depth, using Sony HD HDR-CX6EK, natural daytime illumination, no filter). **Sequence 1b: **Calcareous alga (*Amphiroa*) showing red fluorescence (field at ca. 18 m depth, using Sony HD HDR-CX6EK, natural daytime illumination, red filter, not post-processed). **Sequence 2a: **Unidentified alga under normal viewing conditions (field at ca. 18 m depth, using Sony HD HDR-CX6EK, natural daytime illumination, no filter). **Sequence 2b: **Unidentified alga showing strong red fluorescence (field at ca. 18 m depth, using Sony HD HDR-CX6EK, natural daytime illumination, red filter, not post-processed). **Sequence 3a: **Soft coral (or retracted anemone) under normal viewing conditions (field at ca. 18 m depth, using Sony HD HDR-CX6EK, natural daytime illumination, no filter). **Sequence 3b: **Soft coral (or retracted anemone) showing red fluorescence (field at ca. 18 m depth, using Sony HD HDR-CX6EK, natural daytime illumination, red filter, not post-processed). **Sequence 4a: **Stony coral (*Mycedium*) under normal viewing conditions (field at ca. 18 m depth, using Sony HD HDR-CX6EK, natural daytime illumination, no filter). **Sequence 4b: **Stony coral (*Mycedium*) showing red fluorescence (field at ca. 18 m depth, using Sony HD HDR-CX6EK, natural daytime illumination, red filter, not post-processed). **Sequence 5a: **Stony coral (unknown) under normal viewing conditions (field at ca. 16 m depth, using Sony HD HDR-CX6EK, natural daytime illumination, no filter). **Sequence 5b: **Stony coral (unknown) showing red fluorescence (field at ca. 16 m depth, using Sony HD HDR-CX6EK, natural daytime illumination, red filter, not post-processed). **Sequence 6: **Anemone *Entacmaea quadricolor *showing red fluorescence (field at ca. 18 m depth, using Sony HD HDR-CX6EK, natural daytime illumination, red filter, not post-processed). **Sequence 7a: **Polychaete worm (*Sabellastarte indica*) under normal viewing conditions (field at ca. 18 m depth, using Sony HD HDR-CX6EK, natural daytime illumination, no filter). **Sequence 7b: **Polychaete worm (*Sabellastarte indica*) showing red fluorescence (field at ca. 18 m depth, using Sony HD HDR-CX6EK, natural daytime illumination, red filter, not post-processed). **Sequence 8: **Red reef fluorescence as it appears when viewing the reef through a red mask. Most fluorescent structures are either stony corals or algae (field at ca. 18 m depth, using Sony HD HDR-CX6EK, natural daytime illumination, red filter, not post-processed). Click here for file

Additional File 2 **Red fluorescent fish**. MPG video clip showing fluorescent fish under "normal" viewing conditions *versus *conditions emphasizing red fluorescence. **Sequence 1a: ***Bryaninops natans *(field at ca. 17 m depth, using, natural daytime illumination, no filter). **Sequence 1b: **Several groups of *Bryaninops natans *showing red fluorescence (in the field at 25–18 m depth, using Canon PowerShot G7 and Sony HD HDR-CX6EK under natural daytime illumination through red filter, post-processed to highlight reds). **Sequence 1c: ***Bryaninops natans *individual under changing viewing conditions (laboratory with blue illumination, moving red filter, using Canon PowerShot G7). **Sequence 2a: ***Pseudocheilinus evanidus *in its natural environment (field at 25 m, using Canon PowerShot G7, natural daytime illumination, no filter). **Sequence 2b: ***Pseudocheilinus evanidus *showing red fluorescence (field at 25 m, using Canon PowerShot G7 and Sony HD HDR-CX6EK, natural daytime illumination, red filter, post-processed to highlight reds). **Sequence 3a: ***Ctenogobiops maculosus *at the entrance of its burrow (field at 17 m, using Sony HD HDR-CX6EK, natural daytime illumination, no filter). **Sequence 3b: ***Ctenogobiops maculosus *showing red fluorescent eyes (field at 17 m, natural daytime illumination, red filter, post-processed to highlight reds). **Sequence 4a: ***Ctenogobiops tangaroai *under broad spectrum light (laboratory, using Sony HD HDR-CX6EK, white light form halogen source, no filter). **Sequence 4b: ***Ctenogobiops tangaroai *highlighting red fluorescence under blue light (laboratory, using Sony HD HDR-CX6EK, white light form halogen source filter through scuba-blue filter, recording through red filter, not post-processed). **Sequence 5: ***Eviota pellucida *(*C. tangaroai *in background) highlighting red fluorescence under blue light (laboratory, white light form halogen source filter through scuba-blue filter, recording through red filter, not post-processed). **Sequence 6a: ***Enneapterygius abeli *showing crypsis in large part of spectrum (laboratory, using Canon PowerShot G7, cold light source, no filter). **Sequence 6b: ***Enneapterygius abeli *showing red fluorescence (laboratory, using Canon PowerShot G7, blue light source, red filter, no post-processing). **Sequence 7a: ***Corythoichthys schultzi *showing cryptic colouration (field at ca. 20 m depth, using Sony HD HDR-CX6EK, natural daytime illumination, no filter). **Sequence 7b: ***Corythoichthys schultzi *showing red fluorescence (field at ca. 20 m depth,, natural daytime illumination, red filter, post-processed to emphasize red fluorescence). Click here for file

Additional File 3 **Fish showing behaviour indicative of signalling with red fluorescence**. MPG video clip showing examples of fish behaviours that seem to involve red fluorescence. **Sequence 1**: *Corychthoichthys schultzi *pair interacting, showing use of bright tail fan (field, ca. 20 m depth, using Sony HD HDR-CX6EK, natural sunshine illumination with red filter, post-processed to highlight reds). **Sequence 2a**: *Enneapterygius destai *individual showing extreme crypsis (specimen sits in the middle, lower half, swims briefly to top right at end). **Sequence 2b**: *Enneapterygius destai *individual showing red fluorescence of eye and first dorsal fin. Note visibility of gaze orientation and waving of first dorsal fin caused by excitement (laboratory, blue illumination, using Canon PowerShot G7, red filter). Click here for file
